# The efficacy and safety of tranexamic acid in rheumatoid arthritis patients undergoing simultaneous bilateral total knee arthroplasty: a multicenter retrospective study

**DOI:** 10.1186/s12891-023-06485-9

**Published:** 2023-05-15

**Authors:** Guorui Cao, Shaoyun Zhang, Yixuan Wang, Hong Xu, Songtao Quan, Litao Cai, Wei Feng, Junna Yao, Honglue Tan, Fuxing Pei

**Affiliations:** 1 Department of Knee Surgery, Luoyang Orthopedic Hospital of Henan Province. Orthopedic Hospital of Henan Province, 82 Qiming South Road, Luoyang, Henan Province People’s Republic of China; 2 Department of Orthopedics, The Third Hospital of Mianyang, Sichuan Mental Health Center, Mianyang, Sichuan Province People’s Republic of China; 3 Hunan University of Chinese Medicine, Changsha, Hunan Province People’s Republic of China; 4 Department of Orthopaedic surgery, West China Hospital, Sichuan University, Chengdu, SiChuan Province People’s Republic of China

**Keywords:** Total knee arthroplasty, Simultaneous bilateral, Rheumatoid arthritis tranexamic acid, Blood loss

## Abstract

**Background:**

The efficacy and safety of tranexamic acid (TXA) in reducing blood loss following total knee arthroplasty (TKA) in patients with osteoarthritis have been widely confirmed. However, there is still a paucity of the evidences regarding the effectiveness of TXA in patients with rheumatoid arthritis (RA). The purpose of the study is to explore the efficacy and safety of intravenous TXA on blood loss and transfusion risk following simultaneous bilateral TKA (SBTKA) in patients with RA.

**Methods:**

As a multicenter retrospective study, a total of 74 patients diagnosed with RA who underwent SBTKA were assigned into TXA group (15 mg/kg intravenous TXA before skin incision, n = 50) and control group (no TXA use, n = 24). The primary outcomes were total blood loss (TBL) and intraoperative blood loss (IBL). The secondary outcomes were hemoglobin (Hb) and hematocrit (Hct) drop on postoperative day 3, transfusion rate and volume, ambulation time, length of stay, hospitalization expenses and the incidence of complications.

**Results:**

The mean TBL, IBL and transfusion volume in TXA group were significantly lower than those in control group. The Hb and Hct drop on postoperative day 3 in control group were higher than those in TXA group (*p*<0.05). The similar trend was detected on transfusion rate, ambulation time and length of stay. The incidence of complications and hospitalization expenses did not differ significantly between the two groups (*p*>0.05).

**Conclusions:**

TXA could effectively reduce blood loss, decrease transfusion risk, shorten ambulation time and length of stay following SBTKA in patients with RA, without increasing the risk of complications.

## Background

Rheumatoid arthritis (RA) is an allergic disease, typically manifested as migratory polyarthritis, which often involves bilateral large joints such as knee, shoulder, wrist and hip, resulting in joint pain, deformity and dysfunction [1, 2]. The overall prevalence of RA is 0.32-0.42% in Chinese population [3]. Disease-modifying antirheumatic drugs is the primary treatment means for RA [4]. However, total knee arthroplasty (TKA) is still an effective method to relieve pain, improve knee function and quality of life for patients with advanced-stage RA [5–7].

Simultaneous bilateral TKA (SBTKA) is particularly applicable to patients with RA because RA often affects the knee symmetrically [8]. SBTKA has potential benefits, including a single operation and anesthetic, less recovery time and expense [9, 10]. However, compared with primary TKA, SBTKA is always associated with more blood loss and higher transfusion risk, increasing the incidence of immunologic reactions, transmission of disease, and infection [11–13]. It was reported that allogeneic blood transfusions was more than 60% for patients undergoing SBTKA [14, 15]. Moreover, patients with RA usually experience increased blood loss and transfusions because of severe synovial hyperplasia and knee deformity [16]. Therefore, Blood management in patients with RA following SBTKA is an important challenging for medical workers.

Surgical trauma and hyperfibrinolysis lead to perioperative blood loss in TKA. Tranexamic acid (TXA) is an antifibrinolytic agent, which could competitively inhibit plasminogen activation and plasmin binding to fibrin, thus inhibit the fibrinolysis, then reducing blood loss [17]. In addition, it was reported that TXA could maintain haemostatic threshold about 6 h [18]. Extensive studies have confirmed that tranexamic acid (TXA) could remarkably reduce perioperative blood loss and transfusion risk, promote postoperative rehabilitation without increasing the risk of adverse events in the setting of primary TKA and SBTKA [19–22]. However, the main concerns of previous studies were patients with osteoarthritis and the studies to evaluate the efficacy and safety of TXA in patients with RA were rare. Through extensive reading of relevant papers, we find that only a few literatures report the administration of TXA in patients with RA following primary TKA [23, 24]. The research about TXA application in RA patients undergoing SBTKA is still absent. Thus, we perform this multicenter retrospective study in RA patients following SBTKA to demonstrate (1) whether TXA could diminish blood loss and transfusion risk, (2) whether TXA could decrease ambulation time, length of stay (LOS) and hospitalization expenses, and (3) whether TXA increases the incidence of related complications.

## Methods

### Patients and design

This was a multicenter retrospective study which was approved by local Research Ethics Committee (2012 − 268), and related data was from 15 teaching hospitals in a database. This database was established to evaluate the efficacy and safety of perioperative management for total hip and knee arthroplasty in China in January 2013, sponsored by the Chinese Health Ministry (201,302,007).

SBTKA was defined as bilateral knee replacement performed under a single episode of anesthesia. The patients undergoing SBTKA were identified using International Classification of Diseases, Tenth Revision, Clinical Modification (ICD-10-CM) procedure codes from January 2013 to October 2016. The inclusion criteria included patients diagnosed with RA in Stage III or IV according to the Kellgren-Lawrence classification [25] and patients suffering bilateral TKA simultaneously. Exclusion criteria included surgery because of osteoarthritis, primary TKA, revisions, history of vascular embolism, allergy to TXA and so on. In addition, the patients were excluded if the intraoperative blood loss (IBL) was more than 400 mL during the first knee surgery. Eventually, 74 patients with RA undergoing SBTKA were recruited. The patients were divided into control group (no TXA use, n = 24), and TXA group (15 mg/kg intravenous TXA 5–10 min before the skin incision, n = 50).

### Surgery procedure and perioperative care protocol

All the operations were conducted by senior surgeons using the midline skin incision, medial parapatellar approach and a measured resection technique. The procedures were conducted under general or lumbar anesthesia. The choice of whether to use tourniquet and drainage or not was made by the chief surgeon.

A standardized perioperative care protocol was used in these 15 joint reconstruction centers. All patients receive the routine use of nonsteroidal antiinflammatory drugs from admission to 14 days after surgery. The physiotherapist and doctors instructed patients to perform straight leg raising and walk after surgery. The first ambulation time from bed and operative time were recorded carefully. The combination of physical prophylaxis and chemoprophylaxis for venous thromboembolism were used. Patients received one dose of low-molecular-weight heparin (4000 IU in 0.4 ml, Clexane, Sanofi-Aventis, France) or 10 mg Rivaroxaban (Xarelto, Bayer, Germany) 6–8 h postoperatively, repeating once a day for 10–14 days. Doppler ultrasound examinations were used routinely to detect deep venous thrombosis (DVT). Contrast-enhanced computed tomography scan was performed when pulmonary embolism (PE) was suspected. Transfusions were administered when the hemoglobin (Hb) level was < 70 g/L or 70–100 g/L with anemic symptoms, such as bad mental status, palpitation or shortness of breath not due to other causes.

### Outcome measurements

Demographic characteristics and medical history of the patients were documented preoperatively. The primary outcomes were total blood loss (TBL) and IBL. TBL was calculated based on the formula documented in our previous study [19]. TBL = patient’s blood volume (PBV)×(Hct_pre_-Hct_post_)/Hct_ave_ (Hct_pre_ = the initial preoperative Hct level, Hct_post_ = the Hct on the morning of POD3. PBV = k_1_×height (m)³ + k_2_×weight (kg) + k_3_ (k_1_ = 0.3669, k_2_ = 0.03219, and k_3_ = 0.6041 for men; and k_1_ = 0.3561, k_2_ = 0.03308, and k_3_ = 0.1833 for women, Hct_ave_=the average of the Hct_pre_ and Hct_post_). If either reinfusion or allogeneic transfusion was performed, the TBL was equal to the loss calculated from the change in Hct plus the volume transfuse [26]. IBL was measured as the weight change of gauze and compresses with blood as well as the volume change of aspirators during the whole operation. The secondary outcomes were transfusion rate and volume, Hb and hematocrit (Hct) drop on postoperative day (POD) 3, ambulation and operative time, LOS, hospitalization expenses and the incidence of complications. The LOS was defined as the duration between the day of operation and discharge.

### Statistical analysis

All statistical comparisons were performed using SPSS version 24.0 (SPSS Inc. USA) software. The continuous variables were compared through Wilcoxon Mann-Whitney U test or independent t-test. The categorical variables were compared through Pearson chi-square test or Fisher exact test. Statistical significance was established at *p*-value < 0.05.

## Results

### Patients’ demographics

Totally, 74 patients with RA following SBTKA (24 patients in control group and 50 patients in TXA group) were observed and studied. The baseline characteristics of the patients between the two groups were comparable. The results were summarized in Table 1.

**Table 1:** Baseline Characteristics

Demographics	Control group (n = 24)	TXA group (n = 50)	*p*
Age	60.3 ± 9.8	56.6 ± 15.2	0.209
Gender(male/female)	5/19	11/39	0.909
Height (cm)	159.88 ± 4.89	159.12 ± 7.54	0.656
Weight (kg)	64.1 ± 9.03	64.3 ± 11.8	0.943
BMI(kg/*m*^2^)	25.1 ± 3.48	25.3 ± 3.86	0.805
Range of motion (°)	94.71 ± 13.44	93.70 ± 13.41	0.744
Flexion contractures(n)	18	33	0.289
PBV (mL)	3831.8 ± 410.7	3845.2 ± 621.6	0.913
Anesthesia method (n)			0.196
General anesthesia	22	38	
Regional anesthesia	2	12	
ASA class (n)			0.560
1	8	15	
2	13	32	
3	3	3	
4–5	0	0	
Hypertension (n)	3	11	0.329
Diabetes (n)	3	4	0.845
Osteoporosis (n)	0	4	0.381
Preoperative values			
Hb(g/L)	129.4 ± 17.7	128.6 ± 15.4	0.852
Hct	0.374 ± 0.092	0.373 ± 0.041	0.982
Drainage use (n)	15	34	0.640
Tourniquet use (n)	17	40	0.380

The continuous value was given as the mean and the standard deviation. The categorical values are given as the number of patients. TXA: tranexamic acid; BMI: Body mass index = Weight/Height^2^; PBV: Patient blood volume; ASA: American Society of Anesthesiologists; Hb: Hemoglobin; Hct: Hematocrit

### Blood loss

The mean TBL, IBL and transfusion volume in TXA group (1143.9 ± 717.1 mL, 218.2 ± 199.5 mL, 261.5 ± 218.6 mL) were significantly lower than those in control group (1825.1 ± 747.8 mL, *p*<0.001; 340.0 ± 271.2 mL, *p* = 0.012; 345.3 ± 172.1 mL, *p* = 0.023). Similar trends were detected on Hb and Hct drop on POD3. Moreover, the transfusion rate showed a remarkable decrease in TXA group (26.0%), compared with control group (50.0%, *p* = 0.041) (Table 2).

**Table 2:** Comparison of Blood Loss

Variable	Control group (n = 24)	TXA group (n = 50)	*p*
TBL (mL)	1825.1 ± 747.8	1143.9 ± 717.1	<0.001*
IBL (mL)	340.0 ± 271.2	218.2 ± 199.5	0.011*
Hb drop on POD3 (g/L)	39.4 ± 16.0	28.8 ± 18.4	<0.001*
Hct drop on POD3	0.108 ± 0.091	0.066 ± 0.059	0.002*
Transfusion (n, 100%)	12 (50.0%)	13 (26.0%)	0.041*
Volume of transfusion (mL)	345.3 ± 172.1	261.5 ± 218.6	0.023*

The continuous value was given as the mean and the standard deviation. The categorical values are given as the number of patients. TBL: Total blood loss; IBL: Intraoperative blood loss; Hb: hemoglobin; Hct: hematocrit; POD: postoperative day

### First ambulation time, operative time, LOH and hospitalization expenses

The first ambulation time after surgery was earlier in TXA group, compared with patients in control group ( *p*<0.001). In addition, the operative time was shorter in TXA group (142.5 ± 49.4 vs. 174.8 ± 80.5 min, *p* = 0.036). There was significant difference in LOS (*p* = 0.024) while the difference of hospitalization expenses did not reach statistical significance (*p* = 0.282) between the two groups. Related outcomes were summarized in Table 3.

**Table 3:** Comparison of first ambulation time, operative time, LOH and hospitalization expenses

Variable	Control group (n = 24)	TXA group (n = 50)	*p*
Ambulation time (n, 100%)			<0.001*
≤ 24 h	0 (0.0%)	12 (24.0%)	
24–48 h	6 (25.0%)	8 (16.0%)	
>48 h	18(75.0%)	30 (60.0%)	
Operative time (min)	174.8 ± 80.5	142.5 ± 49.4	0.036*
LOH (day)	11.23 ± 5.25	8.57 ± 4.01	0.024*
Expenses∆	111987.2 ± 22119.9	106017.9 ± 18690.5	0.282

The continuous value was given as the mean and the standard deviation. The categorical values are given as the number of patients. LOH: length of hospital stay

∆Results were presented as Chinese yuan

### Complications

The DVT frequencies were 0 in control group and 2 in TXA group, and the differences were not statistically significant (P = 1.000). In addition, one patient in TXA group and one patient in control group developed wound complication. No PE, periprosthetic fractures, dislocations or cardiac infarction were detected. The incidence of complications was similar between the two groups (Table 4).

**Table 4:** Complications

Complications	Control group (n = 24)	TXA group (n = 50)	*p*
DVT	0	2	1.000
PE	0	0	-
Wound complication	1	1	0.546
Periprosthetic fractures	0	0	
Dislocations	0	0	
Cardiac infarction	0	0	-

DVT: deep venous thrombosis; PE: pulmonary embolism; DVT and wound complication was analyzed by Fisher’s exact test

## Discussion

TKA is a reliable and effective treatment option for end stage RA patients [25, 27]. However, owing to preoperative anemia, great loss of bone mass, severe synovial hyperplasia and knee deformity, RA patients following TKA tend to suffer more blood loss and higher incidence of transfusions compared with OA patients [16]. What is more, the coagulation function between RA and OA patients is different. RA patients is often associated with up-regulated inflammatory factors, such as interleukin 1 and interleukin 6, which could result in the upregulation of procoagulant factors and downregulation of anticoagulation factors [28, 29]. In addition, lots of previous researches confirmed that TXA could significantly decrease perioperative blood loss without increasing the incidence of thrombotic disease in the setting of primary TKA and SBTKA, while the majority literatures focused on OA patients while ignored RA patients [19–21]. Considering the difference of OA and RA, we could not draw definitive conclusion regarding the efficacy and safety of TXA in RA patients. Besides, the patients undergoing SBTKA is confronted with more blood loss and higher risk of complications than primary unilateral TKA [13, 30]. To sum up, the efficacy and safety of TXA administration in RA patients following SBTKA need further investigation.

A very few studies tried to explore whether TXA would decrease perioperative blood loss and whether TXA would add the risk of related complications in patients with RA following TKA [23, 24, 31]. A randomized controlled trail conducted by Kang et al. showed that three doses of postoperative TXA further decreased TBL and HBL without increasing the incidence of adverse events in RA patients following primary TKA [23]. Another study performed by Lei et al. found that TXA use prior to surgery could dramatically diminish blood loss and transfusion requirement, decrease LOS and hospitalization expenses in patients with RA following primary TKA, without increasing the risk of complications [24]. To the contrary, Morse et al. showed TXA did not reduce the risk of transfusion in RA patients after primary TKA. This study was a multivariate analysis and hierarchy of evidence was a little low [31]. To our best knowledge, our study is the first attempt to assess the efficacy and safety of TXA in RA patients in the setting of SBTKA. We show that TXA could reduce blood loss, transfusion rate, Hb and Hct drop without increasing the risk of complications.

Coagulation function between RA and OA patients is different. Some authors demonstrated that RA patients showed higher postoperative blood coagulation condition compared with OA patients, accelerating fibrinogenesis and clot formation [28]. Proteomics of synovial fluid and plasma analysis showed related proteins, which was associated with the activation of the coagulation pathway and subsequent fibrin deposition, were higher in RA patients than that in OA patients [32, 33]. Consequently, RA patients after TKA is possibly associated with higher risk of venous thrombosis. Previous studies have fully demonstrated TXA could not increase the hazard of thrombotic complications for OA patients after primary TKA and SBTKA [19, 34]. Our study firstly evaluate the safety of TXA in RA patients undergoing SBTKA, finding that related complications risk between patients in control group (no TXA use) and TXA group are comparable.

The foundation of achieving enhanced recovery after TKA is effective blood management. Less blood loss means faster recovery. In this study, we found that TXA could markedly shorten ambulation time and LOS after SBTKA. The conclusion was similar to previous papers [17, 20, 24]. In addition, we observed the downward trend of hospitalization expenses in TXA group while the difference was non-significant, may be too little concerned with the number of cases. In summary, TXA application could be as a routine means for RA patients following TKA.

Substantial studies have indicated that multiple doses of TXA or combined use of TXA could further reduce blood loss and transfusions without increasing complication risk following primary TKA [17, 35, 36]. However, the optimal regimen of TXA administration in patients following SBTKA is still controversial. The study performed by Goyal et al. showed that there was not any significant beneficial effect of three doses of TXA as compared to a single dose during SBTKA [37]. A new published study supported this conclusion, suggesting that a single dose of intravenous TXA may be adequate to control excessive blood loss and reduce blood transfusion in SBTKA [38]. Our pervious study found that combined intravenous and topical (intra-articular injection) TXA application failed to get better results in blood loss and transfusion compared with only intravenous TXA application after SBTKA [19]. In other words, most studies considered that a single dose of intravenous TXA is adequate to mitigate transfusion risk as well as reduce blood loss in SBTKA. However, the evidence level of the above studies is a little low and related large sample randomized controlled study is necessary.

It was reported that RA patients presented higher risk of infection following TKA [39]. Some recent studies have proved that the routine use of TXA could decrease the risk of injection in total joint arthroplasty [40, 41]. However, the exact mechanisms by which TXA could contribute to such a reduction need further study. In addition, other methods, such as the application of hemostatic agent Floseal®, have similar effect in decreasing blood loss after primary TKA [42]. Further studies is necessary to address and compare related methods to control bleeding during SBTKA.

The study is carefully designed to lessen bias, such as the nationwide design and matched demographic features, but several limitations are still present. First of all, the sample capacity is small because the patients with RA following SBTKA is less. In addition, considering the difference of various hospitals and surgeon’s experience, the perioperative management methods are not completely consistent. Besides, the information about perioperative coagulation and inflammatory indicators is absent. Last but not the least, the follow-up time is merely one month, which could be too short to evaluate the risk of related complications. Therefore, further studies are requisite.

## Conclusion

TXA could effectively decrease blood loss, reduce transfusion risk, shorten ambulation time and length of stay following SBTKA in patients with RA, without increasing the risk of complications.

## Data Availability

The datasets used and/or analyzed during the current study are available from the corresponding author on reasonable request.
